# Letter from the Editor in Chief

**DOI:** 10.19102/icrm.2021.120202

**Published:** 2021-02-15

**Authors:** Moussa Mansour


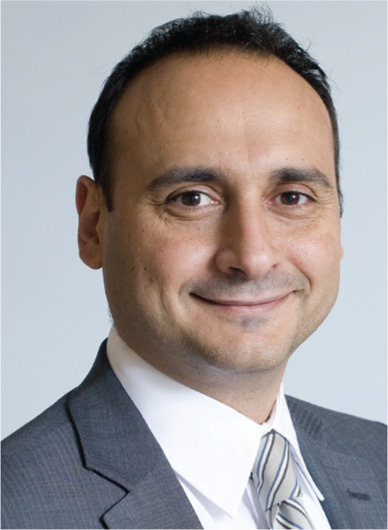


Dear Readers,

The 26^th^ annual meeting of the Atrial Fibrillation (AF) Symposium concluded several weeks ago and, for the first time since its inception in 1994, the Symposium was held virtually. Despite the challenges brought on by the coronavirus disease 2019 pandemic, the meeting was hugely successful, attended by more than 6,000 registrants from around the world. Over three days, more than 100 faculty members presented their innovative, up-to-date work in the form of lectures, debates, panel discussions, live cases, abstracts, and late-breaking clinical trials. Topics ranged from the diagnosis to treatment of AF, and the data presented at the meeting will likely have a significant impact on clinical practice.

One area that attracted significant attention this year was the early treatment of AF. Landmark clinical studies such as the EAST-AFNET trial were presented and discussed in panels. These trials will likely change conventional practices, resulting in wider adoption of early rhythm control in patients with AF. Similarly, presentations and discussions of data from the ATTEST, STOP-AF FIRST, EARLY AF, and CRYO-FIRST trials may lead to catheter ablation being increasingly preferred as first-line therapy over antiarrhythmic medications in patients with AF.

In addition, new technologies for catheter ablation for AF were again extensively covered at this year’s Symposium. In the short term, high-power radiofrequency ablation will likely become the most commonly used ablation technique. This technique incorporates the use of novel catheters containing multiple temperature sensors that provide accurate temperature sensing at the tissue–catheter interface, enhancing the safety of high-power ablation by reducing the chance for steam pop. In the mid-term, the reign of radiofrequency ablation may be challenged by pulsed-field electroporation; data from animal work and first-in-man studies presented at the AF Symposium suggest pristine safety and excellent acute efficacy results are attainable using this novel energy source. While long-term efficacy data remain lacking, once this technology becomes available, the way AF ablation is performed might become radically different.

The rapidly growing area of device-based stroke prevention was also covered in two sessions and a live case. The designs of the eagerly expected CHAMPION and CATALYST studies, which will assess outcomes of left atrial appendage closure versus the use of direct oral anticoagulants, were presented. Once completed, these studies are expected to have a major impact in the field of stroke prevention.

The coverage of these and other topics suggests that progression and development in the field of AF are continuing. Best wishes and I hope that you find the content of this issue of *The Journal of Innovations in Cardiac Rhythm Management* beneficial and educational.

Sincerely,


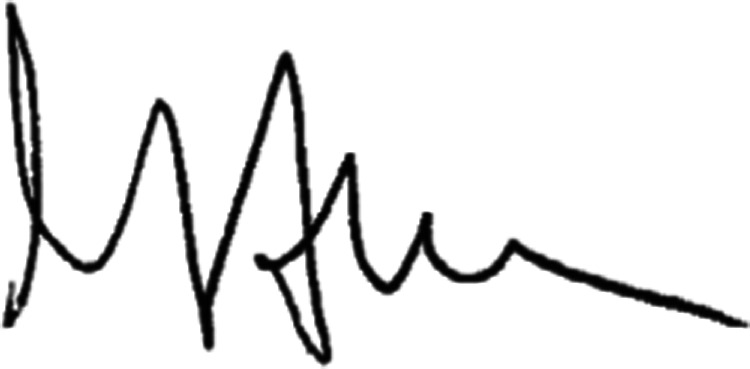


Moussa Mansour, md, fhrs, facc

Editor in Chief

The Journal of Innovations in Cardiac Rhythm Management

MMansour@InnovationsInCRM.com

Director, Atrial Fibrillation Program

Jeremy Ruskin and Dan Starks Endowed Chair in Cardiology

Massachusetts General Hospital

Boston, MA 02114

